# The Evolution of Data Engineering in the Internet of Things

**DOI:** 10.3390/s26185716

**Published:** 2026-09-09

**Authors:** Chia-Hui Wang, Ray-I Chang, Yu-Hsin Hung

**Affiliations:** 1 Department of Computer Science and Information Engineering, Ming Chuan University, Taoyuan City 33348, Taiwan; 2 Department of Engineering Science and Ocean Engineering, National Taiwan University, Taipei 10617, Taiwan; rayichang@ntu.edu.tw; 3 Department of Industrial Engineering and Management, National Yunlin University of Science and Technology, Yunlin County 640301, Taiwan; hungyh@yuntech.edu.tw

## 1. Introduction

The Internet of Things (IoT) has become one of the most influential technological paradigms of the digital era, connecting billions of heterogeneous devices, sensors, machines, and intelligent systems through pervasive communication networks [1,2,3,4]. According to recent industrial reports, the number of connected IoT devices continues to grow rapidly, generating unprecedented volumes of heterogeneous data from smart cities [5,6], intelligent transportation systems, industrial automation, healthcare, environmental monitoring, smart agriculture, and numerous other application domains. While advances in sensing and communication technologies have greatly expanded the capability of IoT systems, the enormous quantity, diversity, and dynamic nature of IoT data have also introduced significant challenges for data acquisition, integration, storage, processing, analysis, security, and intelligent decision-making.

Consequently, Data Engineering for the IoT (DE-IoT) has evolved into a critical interdisciplinary research area that integrates data management, artificial intelligence (AI), machine learning, distributed computing, edge intelligence [7,8], cloud computing, cybersecurity, and intelligent analytics. Rather than focusing solely on efficient data processing, modern DE-IoT research aims to transform massive IoT data streams into actionable knowledge for supporting autonomous decision-making and intelligent services. The emergence of edge computing [9], large-scale AI [10,11,12,13], foundation models [14,15,16,17], and digital twins [18,19,20] has further accelerated this transformation, enabling IoT systems to become increasingly adaptive, context-aware, and autonomous.

During the past several years, research in DE-IoT has expanded considerably beyond conventional sensing and communication infrastructures. Contemporary studies increasingly emphasize intelligent data engineering methodologies capable of addressing data heterogeneity, scalability, privacy preservation, trustworthy AI, cybersecurity, sustainable computing, and real-time analytics. These developments have significantly broadened the scope of IoT research, making data engineering one of the fundamental enabling technologies for next-generation intelligent cyber-physical systems (CPS).

This Reprint Book brings together seven representative studies that collectively reflect the current research landscape of modern DE-IoT. They share several common research objectives, including intelligent data processing, efficient resource utilization, secure and trustworthy system design, and application-oriented intelligent services. This Editorial adopts a broader review-oriented perspective by identifying the major scientific themes emerging from the selected papers and discussing their collective contributions to the development of DE-IoT. Through a thematic synthesis, we highlight the common technological characteristics, emerging applications, and future research opportunities represented by this collection.

The remainder of this Editorial is organized as follows. Section 2 discusses the emerging technical characteristics observed across the selected studies. Section 3 reviews the major application domains and technological trends represented by the collected papers. Section 4 outlines several promising future research directions for DE-IoT, including foundation models, digital twins, trustworthy AI [9,21,22,23], privacy-preserving data engineering, federated learning [24,25], and sustainable AI [26,27]. Finally, Section 5 concludes this Editorial by summarizing and highlighting the continuing evolution of DE-IoT.

## 2. Emerging Special Features of DE-IoT

DE-IoT has evolved well beyond conventional data collection and storage technologies. Contemporary research increasingly focuses on developing intelligent, adaptive, trustworthy, and application-driven data engineering frameworks capable of supporting heterogeneous IoT environments. Although each contribution addresses a different research problem, several common technological characteristics emerge across these studies. These characteristics collectively illustrate the current evolution of DE-IoT and indicate the direction of future research.

### 2.1. AI Has Become the Core Engine of Data Engineering

One of the most significant observations from this collection is the pervasive integration of AI into IoT data engineering. AI has become an essential component throughout the entire data engineering pipeline. In the paper “Long Short-Term Memory Networks’ Application on Typhoon Wave Prediction for the Western Coast of Taiwan” (Contribution 6), the LSTM-based typhoon wave prediction study demonstrates how recurrent neural networks can effectively model long-term temporal dependencies to improve environmental prediction accuracy and extend forecasting lead time for IoT-enabled ocean monitoring systems.

Likewise, in the paper “A Method for Reducing Training Time of ML-Based Cascade Scheme for Large-Volume Data Analysis” (Contribution 5), the modified cascade machine learning framework illustrates that improving computational efficiency through dimensionality reduction can simultaneously reduce training time while maintaining or even improving predictive performance for large-scale biomedical datasets.

Another representative example is the proposed QMVDet framework, “Query-Based Multiview Detection for Multiple Visual Sensor Networks” (Contribution 4), which introduces query-based learning and camera-aware attention mechanisms for multiview visual sensor networks. Instead of uniformly aggregating multiple camera views, the framework intelligently selects the most reliable visual information, significantly improving object detection performance under severe occlusion conditions.

These studies reveal an important trend: AI is no longer simply applied after data collection but is increasingly embedded within data acquisition, feature extraction, data fusion, optimization, and decision-making processes. Although significant progress has been achieved, current AI applications remain largely task-specific. Future research should investigate continual learning, foundation models, multimodal learning, and self-adaptive AI systems capable of supporting heterogeneous IoT environments with minimal human intervention.

### 2.2. Intelligent Data Engineering Is Shifting from Individual Algorithms to System-Level Architectures

A second emerging feature is the transition from algorithm-oriented research toward comprehensive IoT data engineering architectures. In the paper “Design and Implementation of a Novel IoT Architecture for Data Release System Between Multiple Platforms: Case of Smart Offshores” (Contribution 3), the proposed Smart Offshore IoT architecture illustrates this evolution by integrating data acquisition, processing, evaluation, and release into a unified three-layer framework based on dynamic IEC104 [28] configuration, enabling efficient data exchange across heterogeneous offshore platforms.

Similarly, “Methodology for Evaluating Process Mining Tools in IoT Contexts” (Contribution 1) proposes a methodology for evaluating process mining [29,30] tools to emphasize that successful IoT data engineering depends not only on analytical algorithms but also on systematic methodologies capable of transforming sensor-generated event data into actionable process intelligence.

These contributions collectively demonstrate that modern DE-IoT research increasingly considers the entire lifecycle of IoT data, including acquisition, preprocessing, interoperability, analytics, visualization, and operational decision support.

### 2.3. Trustworthiness and Secure Data Engineering Are Becoming Fundamental Requirements

As IoT systems continue expanding into critical infrastructures, ensuring trustworthy communication has become equally important as improving prediction accuracy. In the paper “Context-Aware Trust Prediction for Optimal Routing in Opportunistic IoT Systems” (Contribution 2), the context-aware trust routing framework illustrates this trend by integrating Bayesian inference with Jeffrey’s conditioning to evaluate both social relationships and behavioral reliability when selecting intermediate nodes for opportunistic IoT communications. The proposed framework demonstrates superior delivery ratio, routing efficiency, and trust accuracy compared with existing routing approaches. It introduces intelligent decision-making into IoT networking itself, allowing communication systems to become increasingly context-aware and resilient.

Trust management is gradually evolving from an auxiliary security mechanism into an essential component of intelligent IoT data engineering.

### 2.4. Human-Centered and Application-Oriented Data Engineering

Beyond computational intelligence, the selected papers also demonstrate that IoT data engineering increasingly emphasizes user interaction and application practicality. In the paper “Dual-Message QR Codes” (Contribution 7), the proposed dual-message QR code extends traditional QR technology by enabling two independent messages to be decoded using standard QR readers under different scanning distances, creating innovative human-centered interaction mechanisms without requiring specialized hardware.

Although this contribution differs substantially from the AI-oriented studies, it highlights another important aspect of DE-IoT: effective data engineering must ultimately facilitate efficient communication between intelligent systems and human users. Future IoT applications should not only optimize computational performance but also improve usability, accessibility, and user experience through intelligent interaction mechanisms.

### 2.5. Overall Observations from the Guest Editors

The contributions reveal four closely related characteristics of modern DE-IoT: AI integration across the data pipeline, system-level architectures that connect sensing and computing resources, trustworthy mechanisms for secure and reliable operation, and human-centered information services. These characteristics provide a common framework for interpreting the different application domains discussed in Section 3.

## 3. Emerging Applications of DE-IoT

The contributions cover several application domains in which data engineering transforms heterogeneous IoT data into operational information. This section focuses on how the capabilities identified in Section 2 are manifested in environmental monitoring, healthcare, industrial systems, trustworthy communication, and human-centered information services.

### 3.1. Intelligent Environmental Monitoring and Smart Infrastructure

Environmental monitoring remains one of the most important application domains of IoT. However, recent developments indicate that environmental sensing is gradually evolving from passive observation toward intelligent prediction and autonomous decision support. In Contribution 6, the LSTM-based typhoon wave forecasting study demonstrates how deep learning models can effectively capture long-term temporal dependencies to improve ocean wave prediction, thereby providing more reliable information for maritime safety and disaster prevention. Such predictive capabilities enable proactive decision-making, significantly improving operational resilience in marine environments.

Similarly, the proposed smart offshore IoT architecture extends conventional monitoring systems by integrating data acquisition, evaluation, and dynamic information release across distributed offshore platforms. Contribution 3 proposes a three-layer architecture that provides an efficient infrastructure capable of supporting real-time monitoring and coordinated management of complex offshore systems.

Beyond environmental prediction, Contribution 4 demonstrates the application of intelligent visual sensing in multiview sensor networks. The proposed QMVDet framework uses query-based learning and camera-aware attention to select informative visual features across multiple views, improving object detection under severe occlusion. This example highlights the role of DE-IoT in integrating heterogeneous visual sensing with intelligent perception.

Taken together, these studies illustrate how IoT-enabled sensing can evolve from passive observation toward predictive analysis, coordinated operations, and intelligent perception.

### 3.2. Intelligent Healthcare and Biomedical Data Engineering

Healthcare continues to represent one of the most rapidly expanding application domains for DE-IoT, where massive heterogeneous biomedical data require intelligent processing and accurate decision support. In Contribution 5, the modified cascade machine learning framework demonstrates that efficient feature selection combined with dimensionality reduction can substantially reduce computational complexity while maintaining excellent prediction performance for biomedical datasets. This highlights the importance of scalable data engineering methodologies capable of supporting increasingly large healthcare databases. This study illustrates the growing importance of computational efficiency, making intelligent healthcare systems more suitable for practical deployment in resource-constrained IoT environments.

Healthcare applications can benefit from intelligent data-processing pipelines that integrate wearable sensing, edge intelligence, cloud analytics, and personalized decision support.

### 3.3. Intelligent Industrial Systems and Process Intelligence

Industrial IoT [31,32] has become another representative application domain where efficient data engineering directly influences productivity and operational reliability. The process mining evaluation methodology proposed in Contribution 1 demonstrates that intelligent process analysis can transform sensor-generated event logs into interpretable operational knowledge, enabling engineers to identify bottlenecks, evaluate process conformance, and improve manufacturing efficiency. Importantly, the study also shows that current process mining tools require substantial preprocessing before IoT-enabled data become suitable for intelligent analytics.

Industrial IoT is therefore moving toward data-driven industrial ecosystems in which process intelligence is supported throughout the manufacturing lifecycle. Future studies should investigate real-time process mining, object-centric event modeling, digital twins, and large language models (LLMs) for industrial process analysis.

### 3.4. Secure and Trustworthy IoT Communications

As IoT deployments continue expanding into decentralized environments, communication reliability has become as important as sensing accuracy. In Contribution 2, the proposed context-aware trust routing framework introduces Bayesian inference and Jeffrey’s conditioning to evaluate both behavioral reliability and social relationships during route selection, significantly improving secure communication performance within Social Opportunistic IoT systems. It introduces intelligent decision-making directly into communication infrastructures, illustrating how cybersecurity and AI are becoming increasingly integrated.

The application demonstrates that trust-aware routing can complement conventional communication metrics by incorporating behavioral and contextual information into route selection.

### 3.5. Human-Centered Intelligent Information Services

An important yet often overlooked application of DE-IoT concerns intelligent interaction between IoT systems and human users. The dual-message QR code technology introduced by Contribution 7 demonstrates that innovative information encoding mechanisms can significantly improve data accessibility while remaining fully compatible with existing QR decoding infrastructures. This contribution illustrates that data engineering encompasses not only computational efficiency but also intelligent information presentation and user interaction.

Human-centered IoT services should emphasize intuitive interaction, accessible information presentation, and context-aware delivery so that system intelligence can be effectively used by diverse users.

### 3.6. Overall Observations from the Guest Editors

Across the application domains, the role of DE-IoT differs according to operational requirements: environmental systems emphasize prediction, healthcare emphasizes efficient analysis, industrial systems emphasize process intelligence, communication systems emphasize trust and reliability, and human-centered services emphasize accessible information delivery. The common element is the engineering of data flows and intelligent processing under the constraints of each deployment context.

## 4. Future Perspectives

The selected contributions also reveal several research opportunities that extend beyond the specific methods evaluated in the individual papers. The following directions build on the technical characteristics and application requirements.

### 4.1. Foundation Models for Intelligent IoT Systems

AI has become an important analytical component in DE-IoT. Contributions 4–6 demonstrate the use of machine learning and deep learning for visual perception, large-scale data analysis, and environmental prediction. In particular, Contribution 4 applies query-based learning to multiview visual sensor networks, Contribution 5 improves the efficiency of machine learning-based analysis for large biomedical datasets, and Contribution 6 employs LSTM networks for long-lead-time typhoon wave prediction. These studies illustrate the increasing reliance of DE-IoT on data-driven models and provide a basis for considering more general-purpose AI architectures.

However, the current contributions remain largely task-specific. The rapid development of foundation models and LLMs suggests a potential transition toward unified models capable of handling heterogeneous sensor data, images, event logs, textual information, and operational knowledge. Future research should investigate how such models can be adapted efficiently and reliably to DE-IoT environments.

### 4.2. Digital Twins for Autonomous Data Engineering

Several contributions highlight the importance of continuous data acquisition, process analysis, and system-level information management across distributed IoT environments. Contribution 1 demonstrates how process-mining methodologies can transform IoT-enabled event data into process-level knowledge, while Contribution 3 provides an integrated architecture for data acquisition, evaluation, and release across heterogeneous offshore platforms. Together, these capabilities provide important foundations for future digital-twin systems.

Future DE-IoT systems are expected to extend these capabilities by incorporating digital twin technologies that continuously synchronize physical assets with their virtual counterparts. Such digital representations can support predictive simulation, anomaly diagnosis, maintenance planning, and autonomous operational optimization before actions are executed in the physical world.

Future DE-IoT research is expected to increasingly focus on end-to-end intelligent architectures capable of coordinating distributed sensing devices, edge computing resources, cloud platforms, and AI services within unified CPS. Future studies should investigate standardized data engineering pipelines, process-level data integration, semantic interoperability, digital twins, and autonomous orchestration across heterogeneous IoT infrastructures.

Digital twins are particularly promising for smart manufacturing [20,31,32,33], intelligent transportation, offshore engineering, and critical infrastructures where continuous monitoring and predictive maintenance are essential. Although none of the current papers explicitly develops a complete digital-twin system, several architectures and data-processing capabilities presented in this collection provide building blocks for future implementations.

### 4.3. Trustworthy and Privacy-Preserving Intelligent IoT

As IoT systems become increasingly autonomous, trustworthy data engineering will become equally important as intelligent analytics.

The context-aware trust routing framework proposed in Contribution 2 illustrates how Bayesian reasoning can improve communication reliability through intelligent trust evaluation. Meanwhile, the offshore IoT architecture proposed in Contribution 3 highlights the importance of secure and reliable information exchange across heterogeneous platforms.

Future DE-IoT research is expected to move beyond traditional cybersecurity toward comprehensive trustworthy AI frameworks integrating trust management, explainable AI, privacy-preserving computation, federated learning, and zero-trust architectures. Future systems should incorporate security and trustworthiness throughout the complete data engineering lifecycle.

### 4.4. Edge Intelligence and Autonomous Collaboration

The contributions also highlight the need for distributed and resource-efficient IoT data processing. Contribution 3 presents a multi-platform offshore IoT architecture for coordinating data acquisition, processing, evaluation, and release across heterogeneous platforms, while Contribution 5 demonstrates the importance of reducing computational requirements for large-volume machine learning analysis. Contribution 2 further illustrates the role of intelligent routing in decentralized IoT environments.

These findings suggest that future DE-IoT systems should increasingly distribute learning, inference, and decision-making across edge and cloud resources. Edge intelligence may reduce communication latency and data-transfer requirements while supporting heterogeneous devices with different computational and communication capabilities.

Future research should therefore investigate collaborative intelligence across edge devices, including federated learning, resource-aware model deployment, and adaptive task placement under dynamic network conditions.

### 4.5. Human-Centered and Explainable Intelligent Service

Future IoT applications will increasingly emphasize interaction between intelligent systems and human users. The dual-message QR code in Contribution 7 provides an example of information delivery designed around practical user interaction. Future intelligent services should therefore combine explainable AI, adaptive visualization, natural-language interaction, augmented reality, and personalized decision support to improve user acceptance and operational transparency.

### 4.6. Sustainable and Resilient DE-IoT Ecosystems

Sustainability is not explicitly investigated as a primary objective in the selected contributions; however, several studies point to resource efficiency and operation under constrained environments. Contribution 5 demonstrates that reducing the training time of large-scale machine learning analysis can improve computational efficiency, while Contribution 6 considers an IoT-enabled monitoring environment in which oceanographic sensing and communication are supported by solar-powered buoy infrastructure. These characteristics suggest that resource and energy constraints should receive greater attention in future DE-IoT research.

Future studies should investigate energy-aware sensing, efficient edge inference, communication-efficient distributed learning, green AI [34], and carbon-aware resource allocation. Such approaches should be evaluated together with system reliability, particularly for remote and infrastructure-constrained IoT deployments.

### 4.7. Guest Editors’ Vision

The future directions discussed above are interconnected. Foundation models require reliable and well-engineered data pipelines; digital twins depend on continuous and semantically consistent data; trustworthy AI requires security, provenance, and privacy mechanisms; edge intelligence requires efficient distributed processing; and sustainable IoT requires joint optimization of computation and communication.

An important research opportunity is therefore the development of integrated DE-IoT architectures that coordinate these capabilities across the complete data lifecycle while preserving reliability, efficiency, privacy, interpretability, and resilience.

## 5. Conclusions

This Reprint Book provides a diverse view of the continuing development of Data Engineering for the Internet of Things. They cover intelligent prediction, efficient machine learning, visual sensing, offshore IoT architectures, process mining, trust-aware routing, and human-centered information interaction. Although their technical objectives differ, the studies collectively show that DE-IoT increasingly concerns the transformation of heterogeneous data into reliable and useful intelligence under practical system constraints.

Four observations emerge from the collection. First, AI is becoming integrated into multiple stages of IoT data processing. Second, system-level engineering is increasingly important because useful IoT services depend on coordinated sensing, communication, computation, analytics, and data management. Third, trustworthiness and security should be treated as properties of the data lifecycle. Fourth, human-centered interaction remains important because intelligent information must ultimately be understandable and usable by its intended recipients.

The application examples further show that DE-IoT is not restricted to a single sector. Environmental monitoring, biomedical analysis, industrial process intelligence, trustworthy communication, and interactive information services impose different requirements on data engineering. This diversity reinforces the need for architectures that can adapt their processing, communication, and analytical strategies to each deployment context.

Looking forward, foundation models, digital twins, edge intelligence, federated learning, privacy-preserving computation, trustworthy AI, human–AI collaboration, and sustainable computing provide promising directions for extending the capabilities observed in the selected studies. The key challenge is to integrate these technologies without sacrificing reliability, efficiency, privacy, interpretability, or operational resilience. More broadly, data engineering is increasingly becoming a strategic foundation through which IoT systems perceive environments, derive knowledge, coordinate actions, and support human decisions. Continued integration of AI, distributed computing, trustworthy mechanisms, and human-centered design can therefore strengthen DE-IoT as a foundation for future intelligent and resilient CPS.
